# Extremely rare mucinous adenocarcinoma of the small bowel causing bilateral metastatic Kukenberg tumors of the ovaries: A case report

**DOI:** 10.1097/MD.0000000000040397

**Published:** 2024-11-01

**Authors:** Lichao Zhu, Ye Liu, Shuai Chen, Guanghua Yang, Changyou Wang

**Affiliations:** a Affiliated Hospital of North China University of Science and Technology, Gastrointestinal Oncology Treatment Center, Tangshan, Hebei, China; b Affiliated Hospital of North China University of Science and Technology, Breast Disease Treatment Center, Tangshan, Hebei, China.

**Keywords:** Krukenberg tumor, ovarian metastases, ovarian tumors, small bowel cancer

## Abstract

**Rationale:**

Small bowel adenocarcinoma (SBA) is an extremely rare tumor that is not fully understood, SBA accounts for less than 5% of gastrointestinal cancers, Krukenberg tumors account for a lower proportion of all ovarian tumors, close to 2%. Stomach is the most common primary site of Krukenberg tumor. The phenomenon of bilateral ovarian Kukenberg tumor caused by implantation and metastasis of small bowel cancer is extremely rare, with few literature reports and limited clinical diagnosis and treatment data. We present a case of a 55-year-old woman with bilateral Kukenberg’s tumor caused by small bowel cancer implantation and share our views on the diagnosis and treatment of this case.

**Patient concerns:**

A 55-year-old woman presented with vaginal bleeding and persistent lower abdominal pain after fatigue 10 days ago. Pelvic ultrasound at a local hospital revealed 2 solid masses in her pelvis, and she came to our hospital for further diagnosis and treatment. The results of colonofiberscope examination and histopathological examination confirmed intramucosal adenocarcinoma in the small intestine.

**Diagnoses:**

The results of colonofiberscope examination and histopathological examination confirmed intramucosal adenocarcinoma in the small intestine. Contrast-enhanced computed tomography showed multiple cystic space-occupying lesions in the pelvic cavity, and the possibility of ovarian tumor was considered.

**Interventions:**

Radical treatment of right half colon cancer and pelvic mass resection were performed under general anesthesia. Combined with intraoperative and postoperative pathological examination, the diagnosis was mucinous adenocarcinoma of the small intestine stage IV (pT4N1M1). Bilateral ovarian metastasis, metastatic cancer (3/19): lymph nodes around the small intestine (3/12), lymph nodes around the colon (0/7).

**Outcomes:**

He is currently receiving chemotherapy, the chemotherapy regimen is XELOX regimen. The specific drugs were oxaliplatin and capecitabine.

**Lessons:**

SBA is often difficult to diagnose due to few specific symptoms and is usually detected at stage IV. Bilateral ovarian Kukenberg tumor caused by small bowel cancer implantation metastases is extremely rare, and clinicians must be vigilant for women with fewer specific symptoms of gastrointestinal discomfort and conduct further diagnostic studies to avoid delayed diagnosis and treatment.

## 1. Introduction

Krukenberg tumor (KT) is a rare metastatic ovarian cancer named after Friedrich Ernst Krukenberg, a German physician, who first described 5 cases of KT, histologically characterized as a signet ring adenocarcinoma originating predominantly from the gastrointestinal tract, with 35 to 40 years of age being the most common.^[1]^ Most patients present with bilateral ovarian involvement and have a very poor prognosis with a median survival of only 14 months.^[2]^ The stomach is the most common site of origin, followed by the colon, and the small intestine is extremely rare.^[3]^

KT is characterized by stromal involvement, mucin production, tumorigenic imprinted cells, and sarcomatoid proliferation. Here, we present a case of atypical KT, a giant mucinous adenocarcinoma of the small intestine leading to bilateral ovarian KTs and highlight the importance of timely diagnosis and treatment of the disease. This case is reported according to CARE criteria.

## 2. Case report

A 55-year-old female from Hebei Province, China, was admitted to her local hospital on April 29, 2024, due to persistent lower abdominal pain due to vaginal bleeding after fatigue 10 days ago, but the abdominal pain was bearable without adequate attention, and the abdominal pain worsened after a cold 1 day ago. Pelvic ultrasound in local hospital showed that 2 cystic solid masses were observed in the pelvic cavity, the larger one at the upper right of uterus was about 12.75 × 9.13 cm, and the smaller one at the upper left front of uterus was about 4.58 × 3.93 cm, with clear boundary and regular shape. Strong solid echoes can be seen in the inner margin of the mass capsule, and the meshed and flocculent echoes can be seen in the capsule cavity.

**Figure 1.**
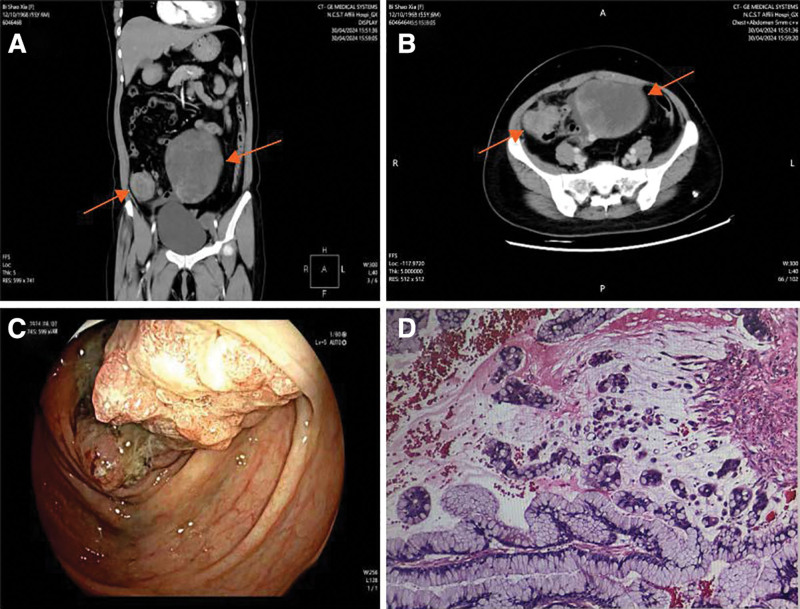
Preoperative imaging and colonofiberscope. Contrast-enhanced CT shows (A) multiple irregular hypodense masses were seen in the pelvis in the coronal position. The ileocecal mass as shown by the arrow on the left side of (A) has a narrowed bowel lumen and thickened bowel wall with circumferential enhancement on enhancement scan. The left ovarian mass is shown as the right arrow in (A), and there are strips of obvious enhancement in the left ovary, which is about 12.2 cm × 8.7 cm × 12.7 cm; contrast-enhanced CT shows (B) the thickening of the intestinal wall in the ileocecal region is detected in the transverse view, as shown by the left arrow in (B), and there is obvious enhancement in the enhancement scan. The fat space around the ileocecal wall was increased in density, and a nodular focus with a diameter of about 1.3 cm was seen with moderate enhancement, which was closely related to the left ovarian mass. Colonofiberscope shows (C) an irregular mass growing into the lumen of the bowel was seen in the ileocecal region, which filled up the lumen completely, and the surface of the nodularity was not flat. The surface of the mass was ulcerated and bled easily on contact, and a biopsy was taken endoscopically for pathological examination. Pathological examination of biopsy under endoscopy (D) under the microscope, the cancer cells were columnar, with dark staining and large nuclei.

For further treatment, the patient came to the Affiliated Hospital of North China University of Science and Technology for treatment. Contrast-enhanced computed tomography (CT) showed multiple solid cystic space-occupying lesions in the pelvic cavity, and the possibility of ovarian tumor was considered. Neoplastic lesions with enlarged peripheral lymph nodes were also seen in the small intestine (Fig. 1A and B). The lower digestive tract angiography showed space-occupying lesions in the small intestine with a high probability of malignancy. Subsequently, we performed colonofiberscope examination. Under the microscope, the small intestine showed an irregular mass growing inside the intestinal cavity, completely filling the intestinal cavity, and the surface was uneven like nodules. The surface of the tumor was ulcerated and bleeding was easy to contact. Histopathological examination revealed intramucosal adenocarcinoma in the small intestine (Fig. 1C and D). The laboratory tests showed that glycogenic antigen 125 was 41.430U/mL, carcinoembryonic antigen 153 was 11.010 U/mL, glycogenic antigen 199 was 23.440 U/mL, and glycogenic antigen 724 was 131.100 U/mL. Carcinoembryonic antigen was 7.9 ng/mL, neurospecific enol was 14.000 µg/L, premenopausal Rome index was 13.421%, postmenopausal Rome index was 26.176%, and human papillomavirus 66 was positive. Other laboratory tests showed no significant abnormalities.

**Figure 2.**
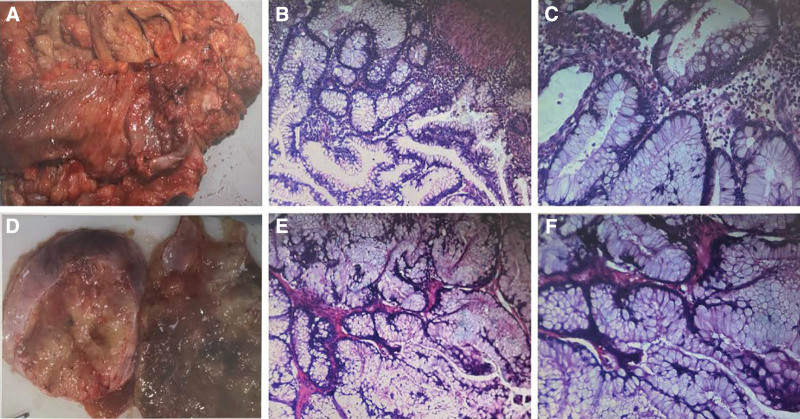
Intraoperative pathology (A–C) for radical resection of right colon cancer and intraoperative pathology (D–F) for pelvic mass resection. Rough diagram of ileocecal mass (A) The swelling is hard, about 6 × 5 × 3 cm in size, and the mass is soaked into the serous layer. Hematoxylin-eosin staining, original magnification ×10 (B), original magnification ×20 (C), cancer tissue is composed of irregular glandular ducts, and the cancer cells are columnar, with large nuclei, deep staining, disordered arrangement, and pathological nuclear division. Gross diagram of bilateral ovarian masses (D). The size of the left ovarian mass is about 8 × 8 × 5 cm, the size of the right ovarian mass is about 20 × 20 × 10 cm, and the bilateral ovarian mass has a complete capsule and a smooth surface. Hematoxylin-eosin staining, original magnification ×10 (E), original magnification ×20 (F), the same results can be seen in the pathology of the ileocecal mass, the cancer tissue is composed of irregular glandular ducts, the cancer cells are columnar, the nucleus is large, and the staining is deep.

The patient had adenocarcinoma in the small intestine with pelvic mass. The indications for surgery were clear and there were no surgical contraindications. Radical resection of right half colon cancer plus pelvic mass resection was performed under general anesthesia on May 15, 2024. Intraoperatively, the mass in the small intestine was hard in texture and about 6 × 5 × 3 cm in size. The mass penetrated the serous membrane and reached the right abdominal wall (Fig. 2A). The size of the left ovarian tumor was about 8 × 8 × 5 cm, and the size of the right ovarian tumor was about 20 × 20 × 10 cm. The envelope of the bilateral ovarian tumor was complete and the surface was smooth (Fig. 2D). Rapid pathological examination was performed during the operation, and the results showed that the mucinous adenocarcinoma in the small intestine (Fig. 2B and C) was composed of irregular glandular ducts. The cancer cells were columnar, with large nuclei, deep staining, and disordered arrangement, and pathological nuclear division was visible. Both ovaries are borderline mucinous tumors with 5% sig-ring cell carcinoma (Fig. 2D and E). After careful pathological observation, it was finally determined that the bilateral ovarian tumors were derived from mucinous adenocarcinoma of the small intestine. No obvious abnormalities were found in the left and right fallopian tubes. Metastatic cancer was found in lymph nodes (3/19): lymph nodes around the small intestine (3/12) and lymph nodes around the colon (0/7). No abnormalities were found in other organs or tissues during the operation. After the operation, the patient was given anti-inflammatory treatment, inhibition of gastric acid secretion, fluid supplementation, pain relief, parenteral nutrition, and other supportive treatments. Immunohistochemical results showed CDX-2(+), SATB2(+), cytokeratin 7 (CK7−), cytokeratin 20 (CK20+), PAX-8(−), estrogen receptor−. Special dyeing result: elastic fiber dyeing (+).

Combined with intraoperative and postoperative pathological examination, she was diagnosed with stage IV mucinous adenocarcinoma of the small intestine (pT4N1M1), bilateral ovarian metastasis, metastatic cancer (3/19): lymph nodes around the small intestine (3/12), and lymph nodes around the colon (0/7). The wound healed well after operation, with no inflammation and bad performance such as redness, swelling, and rupture. At present, the patient has no obvious discomfort. The patient was receiving chemotherapy, the chemotherapy regimen was XELOX regimen, and the specific medication was oxaliplatin on the first day with 130 mg/m^2^ body surface area, and capecitabine on the first to 14th day with 1000 mg/m^2^ body surface area, twice a day, once after breakfast and dinner, and then rest for a week, that is, a complete chemotherapy cycle.

## 3. Discussion

Small bowel adenocarcinoma (SBA) is a very rare tumor, SBA accounts for less than 5% of gastrointestinal cancers,^[4]^ although the small intestine occupies an extremely high proportion of the length of the intestine and the surface area of the digestive tract, the diagnosis of SBA is often difficult due to specific symptoms. So it usually finds the disease at stage IV. SBA has a poor prognosis, with a 5-year life expectancy of 14% to 33%.^[5]^

The overall incidence of SBA is on a slow upward trend,^[6]^ alcohol consumption, smoking, and obesity are associated with an increased risk of this tumor, and diet plays an important role because higher rates of SBA are found among consumers with lower intakes of carbohydrates, red meat and coffee, fruits, and vegetables. Therefore, dietary risk factors in SBA and colorectal cancer are similar in humans.^[7,8]^ The diagnosis of SBA is usually insidious, and the most common symptoms are abdominal pain, weight loss, indigestion, diarrhea, nausea, vomiting, bloating, fatigue, and gastrointestinal bleeding. SBA can lead to specific complications, depending on tumor location, including jaundice, obstruction, and perforation. These symptoms may be similar to other more common benign conditions such as cholelithiasis, cholangitis, pancreatitis, inflammatory bowel disease, or appendicitis, which could explain that the average interval between symptoms and diagnosis can be close to 2 years or so due to the limited sensitivity of routine radiographic imaging. It may also lead to delayed diagnosis.^[9,10]^ Conventional imaging with CT or magnetic resonance should be used for staging purpose.^[11,12]^

KTs accounted for a relatively low proportion of all ovarian tumors, nearly 2%. Almost all KTs were metastatic, and only a few rare primary occult tumors were found. Stomach is the most common primary site of KTs, while tumors originating in small intestine are extremely rare.^[13,14]^ However, it has also been observed that the incidence is higher not from the stomach, but from the colon.^[15]^ The overall prognosis of KTs is poor, given the delayed diagnosis and more advanced presentation. The overall mortality rate for patients with KTs is very high. The authors of almost all reported cases highlight the pessimistic outcome of this tumor. Most patients die within 2 years, with a median survival of 14 months.^[16]^

Bilateral Krukenberg ovarian tumors may be asymptomatic or present with nonspecific gastrointestinal signs and symptoms, such as nausea, vomiting, abdominal or pelvic pain, bloating, and ascites. The classical gross pathological features of KTs of both ovaries are bilateral, asymmetrically enlarged ovaries with convex surfaces. Radiologically, bilateral Krukenberg ovarian tumors typically present as bilateral, irregular, hyperechoic solid patterns with well-defined cystic regions that produce prominent vascular signals along the cyst wall. CT usually shows a solid ovarian mass, which is different from primary ovarian cancer.^[17]^

Immunohistochemical assessment may be helpful in differentiating primary ovarian cancer from metastatic ovarian cancer. Cytokeratin 7 and Cytokeratin 20 (CK7 and CK20) immunophenotypes are the most commonly used methods for analysis. Primary ovarian cancer is almost always immunoreactive to CK7 (90%–100%), but usually not to CK20. In contrast, metastatic small bowel cancer is less positive for CK7, but positive for CK20 in most cases. Colorectal adenocarcinoma is usually negative for CK7, but in most cases positive for CK20. Tumors that metastasize from the appendix are usually positive for CK20, but CK7 is also found in 50% of cases. Thus, the CK7+/CK20− immunophenotype favors primary ovarian cancer, while the CK7−/CK20+ or CK7+/CK20+ immunophenotypes (especially CK20+) favor metastatic gastrointestinal cancer.

Surgery is the primary treatment for small bowel cancer and the only cure option for local area disease. The type of resection depends on the location of the tumor. For tumors located in the jejunum or ileum, segectomy is recommended, including lymph node dissection and jejune-jejunum or ileoileostomy, while for tumors involving the last loop of the ileum or ileocecal valve, segectomy is recommended. Ileocecectomy or right hemicolectomy, including loop ilectomy and ligation of ileocolic arteries, to allow for adequate lymph node dissection.^[18]^

Serum CA125 levels may be elevated in KT patients before surgery, but subsequently decreased after tumor resection.^[19]^ Based on this observation, serum CA125 levels can be used for postoperative follow-up to evaluate complete tumor resection and for early detection of ovarian metastases in patients with a history of primary adenocarcinoma, especially in the gastrointestinal tract. Serum CA 125 levels are also helpful in predicting prognosis. In a study investigating serum CA125 levels in KTs, it was found that compared to patients with CA125 levels below 75 U/mL, patients with preoperative serum CA125 level greater than 75 U/mL have a low 5-year survival rate,^[20]^ and CA125 is also the only important prognostic marker for non-gynecological ovarian cancer.^[20]^ Peritoneal involvement, simultaneous treatment, ascites, and elevated serum CEA levels seem to be adverse prognostic factors for KT and may affect the management of patients.^[21]^

Due to insufficient guidelines for the treatment of bilateral ovarian metastases caused by small bowel cancer. The best chance to increase patient survival is metastasectomy in surgical candidates followed by palliative chemotherapy in metachronous tumors and both resection of primary tumor and metastasectomy followed by palliative chemotherapy in synchronous tumors.

## 4. Conclusions

An uncommon illness that progresses quickly is the KT. The prognosis of KT, an uncommon illness that advances rapidly, can be improved with the right diagnosis and treatment. In determining the source of metastatic adenocarcinoma, immunohistochemistry staining is helpful. Given their rarity, a national registry should be established to gather data on patients with KTs to enhance treatment outcomes and diagnosis.

## Acknowledgments

We are grateful to the patient and her husband who gave their consent for her image and clinical information to be published. Informed consent from the patient has been obtained for publication of the case.

## Author contributions

**Conceptualization, writing—original draft:** Lichao Zhu.

**Methodology:** Lichao Zhu, Ye Liu.

**Project administration:** Ye Liu.

**Data curation:** Shuai Chen.

**Formal analysis:** Guanghua Yang.

**Funding acquisition, writing—review & editing:** Changyou Wang.
